# Two plants improve stress response of a subterranean herbivore by downregulating amphetamine addiction pathways

**DOI:** 10.3389/fvets.2023.1342630

**Published:** 2024-01-12

**Authors:** Feiyu Zhang, Yuchen Tan, Zhiyuan Cai, Kang An, Yongjie Liu, Junhu Su

**Affiliations:** ^1^Southwest Survey and Planning Institute of National Forestry and Grassland Administration, Kunming, China; ^2^College of Grassland Science, Key Laboratory of Grassland Ecosystem (Ministry of Education), Gansu Agricultural University, Lanzhou, China; ^3^Gansu Agricultural University-Massey University Research Centre for Grassland Biodiversity, Gansu Agricultural University, Lanzhou, China

**Keywords:** anti-stress, behavior, captivity environment, plateau zokor, stress

## Abstract

**Introduction:**

Captivity serves as the primary method for enhancing animal survival and productivity. However, the stress induced by confinement can hinder animal growth and reproduction. The administration of drugs to captive animals can effectively regulate their stress response and can also be used inartificial breeding, reproduction, and experimental animalization of wild species. The plateau zokor (*Eospalax baileyi*), a subterranean herbivore, experiences significant stress during the captive process owing to its unique habitat.

**Methods:**

In our study, we utilized *Radix astragali* (RA) and *Acanthopanax senticosus* (AS) extracts to intervene in the stress response of plateau zokors.

**Results:**

Our findings demonstrated that RA and AS treatment considerably improved food intake and reduced weight loss, stress-related behavior, and stress hormone levels in plateau zokors. Furthermore, the excitatory pathway of amphetamine addition in the hypothalamus was suppressed by RA and AS treatment, acting through the *Grin* and *Prkc* gene families. Notably, after RA treatment, the extracellular matrix-receptor interaction pathway, enriched by the *Col1a1/3a1/1a2/6a1* gene, was significantly upregulated, potentially enhancing the immune function of captive plateau zokors.

**Discussion:**

In conclusion, our research demonstrates that RA and AS treatment can effectively alleviate the stress response of plateau zokors in captive environments. The downregulation of the excitation pathway and upregulation of the immune pathway offer valuable insights into the response and potential mechanisms of plant-based drugs in mitigating animal stress.

## 1 Introduction

Captivity is the primary method used to enhance the survival and reproduction of animals. Implementing captive breeding allows for controlled reproduction, which is of immense significance in improving artificial breeding, experimental animalization, and overall reproductive success of wildlife (1). However, the process of animal captivity exposes them to various stressors, some of which are nearly unavoidable, including high temperatures, cold conditions, overcrowding, noise, and human interference (2). The captive environment often lacks complexity and opportunities for interaction with the surrounding environment or the outside world, resulting in psychological and physiological stress for the animals (3). The impact of these stressors during captivity can be severe, leading to mental stress, endocrine disorders, decreased immunity, and the development of various diseases (4). These adverse effects are manifested through interruptions in the reproductive cycle, behavioral abnormalities, inhibited growth rates, weight loss, and, in severe cases, even death (5). Additionally, enduring captivity can cause animals to experience sensory feedback issues, motor control abnormalities, vomiting, dizziness, and disorientation (6). Artificial lighting devices create sharp contrasts between bright and dark areas, while temperature fluctuations further elevate the stress levels of animals (7). Therefore, it is crucial to prioritize the reduction of stress in animals during captivity, enhancing their ability to cope with stress, alleviate pain, improve vitality and productivity, and ultimately promote the successful utilization of wild animals.

There are two main methods to reduce the stress of captive animals: replicating the natural environment and drug intervention. In addition to replicating the natural environment, one effective method of reducing animal stress in captivity is drug intervention, which is both simple and economically feasible (8). Chemical drugs, such as sedatives and anesthetics, are commonly used to alleviate stress in animals. These drugs are administered orally or through injections before captivity to calm the animal’s nervous system and reduce stress reactions (9). However, such drugs can be highly toxic, and excessive use can increase the risk of death. Additionally, drug accumulation can occur, posing a threat to the animal’s health and safety (10). Furthermore, the use of drugs in animals can lead to physical dependence (11). While they may initially alleviate stress, they can also lead to severe withdrawal symptoms, making them unsuitable for long-term stress reduction in captive animals. Therefore, it is crucial to develop and utilize new drugs specifically designed to alleviate the stress response in captive animals.

The use of medicinal plant extracts, such as those employed in Traditional Chinese Medicine, has gained popularity due to their safety, efficacy, and cost-effectiveness (12). Research has shown that administering certain doses of medicinal plant extracts can counteract the damage caused by stress (13). For example, *Cordyceps sinensis* fruit extract has been found to alleviate stress and reduce damage to the antioxidant enzyme system in stressed rats (14). Chamomile root and leaf extracts also exhibit significant anti-stress and anti-anxiety activities. These medicinal plant extracts can stabilize stressed animals and alleviate environmental stress. However, previous studies have primarily focused on the biochemical levels of captive animals, making it difficult to fully understand how medicinal plant extracts treat stress responses in wild animals (15). The neuroendocrine theory suggests that animals under stress mobilize various organs and tissues to respond to stressors through the neuroendocrine system (16). The body maintains coordination and balance of physiological and biochemical processes through complex neurohumoral regulation, establishing a new stable state (17). The central nervous system, particularly the cerebral cortex, plays an integral role, while various axes, such as the sympathetic adrenal medullary system and hypothalamic–pituitary–adrenal cortex (HPA) axis, have executive functions (18). The hypothalamus is the central center in regulating stress. With advancements in molecular biology and high throughput sequencing technology, applying these new technologies to identify new anti-stress pathways of different drugs, alleviate specific stress levels in different animal groups, and improve animal survival ability is crucial to promote wildlife experimentation and subsequent utilization.

The plateau zokor (*Eosplax baileyi*) is a unique underground rodent found in the Qinghai Tibet Plateau, residing in underground nests throughout the year with limited migration abilities (19). It holds significant ecological research value due to its remarkable adaptation to harsh conditions, including cold temperatures, low oxygen, and high humidity (20). Moreover, its unique ability to thrive in hypoxic and humid environments makes it an ideal model for physiological, medical, and ecological research (21). However, conducting controlled indoor experiments and breeding of plateau zokors in captivity remains a considerable challenge. These animals strongly prefer their natural plateau habitat and struggle to survive under indoor breeding conditions, resulting in often fatal stress. Consequently, the underground-dwelling plateau zokor serves as an excellent model for studying the potential stress-alleviating effects of medicinal plants in wild animals. In this study, we selected *Radix astragali* (RA) and *Acanthopanax senticosus* (AS) as the plant components for investigation. RA is widely used for medicinal purposes and contains *Astragalus* polysaccharides, while AS possesses phenolic hydroxyl groups (22, 23). Previous research has demonstrated that RA and AS exhibit antioxidant, anti-fatigue, stress-relieving (24), immunoregulatory, anti-inflammatory, and anti-tumor activities. These properties are closely related to the adaptability, anti-stress, and anti-inflammatory characteristics of medicinal plants (25, 26). Studies have shown that RA treatment significantly mitigates environmental stress in captive chickens (27). Furthermore, the use of AS in treating mice with Parkinson’s disease has shown to reduce climbing frequency and increase autonomous exercise (28). Based on these findings, we anticipate that the extracts from these two medicinal plants will improve the stress behavior and physiological conditions of plateau zokors.

This study investigated the impact of RA and AS treatments on the behavior, growth performance, heart rate, hormone levels, and mortality rate of plateau zokors. RNA sequencing technology was utilized to analyze the characteristics of the hypothalamic transcriptome in the treated individuals. Additionally, bioinformatics was employed to analyze the differentially expressed genes (DEGs), their functions, potential pathways, and to screen candidate regulatory transcription factors. The research aimed to determine whether these medicinal plant extracts can enhance the behavior, physiological status, and survival rate of plateau zokors under stress conditions. Furthermore, the study aimed to identify pathways and transcription factors associated with drug-induced stress relief, and the findings could inform future research on captive stress and drug development in wild animals.

## 2 Materials and methods

### 2.1 Animals

In September 2019, we captured 96 plateau zokors (48 males and 48 females) safely using live trapping cages during the non-breeding season. These test animals were all from Minxian County, Gansu Province (103°41′23″ E, 34°07′34” N). We housed the animals indoors in 50 × 40 × 40 cm storage boxes with ventilated lids. Each box was filled with clean soil, a PVC pipe measuring 8 cm in diameter, and the animal rearing room was kept in complete darkness to simulate the underground environment of the zokor. Each zokor was housed in an individual cage and maintained under standard conditions of 18 ± 2°C and 45–55% relative humidity. The animals had access to fresh sweet potatoes (*Ipomoea batatas*) and carrots (*Daucus carota*), which are the preferred foods of zokors. After 3 days of acclimatization in the indoor environment, we conducted experiments to assess the behavior of the animals and measure other physiological and biochemical indices, minimizing interference from other factors with the results of drug experiments (29).

### 2.2 Experimental treatments

AS and RA roots were obtained from the Lanzhou Yellow River Herb Market (Lanzhou, China) and were identified by Professor Junhu Su of the College of Grassland Science at Gansu Agricultural University. The dried roots were crushed, soaked in distilled water, boiled, and filtered twice. The two filtrates were combined and then concentrated by rotary concentration evaporation under vacuum at 60°C (Christ RVC 2–25 CD plus, Osterode, Germany) to obtain the RA and AS root concentrates. After further freeze-drying, the concentration was adjusted to 10 g/mL, and the extracts were stored at 4°C until further analysis (30).

### 2.3 Methods

#### 2.3.1 Experimental grouping

After 3 days of acclimatization, the plateau zokors were randomly divided into three groups: control (CON), RA, and AS, each comprising 32 zokors (16 males and 16 females). Each plateau zokor was individually numbered. The sample size was chosen to ensure the efficacy of detecting the effects of interest (31, 32). Based on previous experiments and experience, the volume of drug administered was 0.1 mL per 100 g of body mass, taking into account the tolerance level of the wild animals and reducing the stress induced by manual drug administration. During the experimental period, each treatment group received RA and AS aqueous extracts at 09:00 by oral gavage for 7 days, while the CON group was administered an equal volume of distilled water (Supplementary Figure S1). At the end of the behavioral experiment, all animals were euthanized, and blood samples were collected. The hypothalamus was removed after dissection for further analyses. The tissue samples were immediately frozen in liquid nitrogen and then stored at −80°C for subsequent use.

#### 2.3.2 Measurement of growth performance

The growth performance of plateau zokors that remained healthy and alive for 7 and 14 days was assessed based on weight variation and food intake, respectively. Body weight was measured at 1, 8, and 14 days, and the difference in body mass before and after the experiment was used to calculate the change in body mass at 7 and 14 days.

The amount of food consumed by the animals was calculated by recording the weight of the feed provided daily and weighing the remaining food the next day at the same time as each feeding. The daily food intake of each plateau zokor was then determined. The food intake for 7 days represented the total intake from day 1 to 7, and the food intake at 14 days represented the total intake from day 8 to 14.

#### 2.3.3 Heart rate measurement

Heart rate detection was conducted according to the method described by Qu (33). Recordings were made in a quiet, noise-free room, and the heartbeat of each animal was recorded for 2 min using a recording stick (Sony Stereo Digital Recorder; ICD-SX2000). The audio recordings were processed using Adobe Audition, and waveforms were plotted. The data were analyzed by recording a stable heartbeat for 15 s and calculating the number of heart beats per minute.

#### 2.3.4 Behavioral measurements

To simulate a dark – light environment, a glass open-field test experimental chamber measuring 100 × 100 × 50 cm was used. The box was covered with black stickers, and the bottom of the experimental chamber was divided into 20 × 20 cm square lattices. The central lattices constituted the middle nine lattices of the chamber, while the remaining 16 formed the outer perimeter area (Supplementary Figure S2). Behavioral measurements were conducted in a dark environment with no disturbance or odor. Each plateau zokor was placed in the central compartment at the start of the experiment. The behavioral performance of the plateau zokors was recorded for 5 min using an infrared video webcam (ZKXC.TD136U2RZT, Zhongke Electric Co., Ltd., Shenzhen, China), with one measurement performed for each animal. Between individual zokors, the inside and outside of the experimental chamber were thoroughly cleaned with 25% alcohol to eliminate odors. After the alcohol completely evaporated, the next zokor was assessed.

Behavioral videos were analyzed and processed using BORIS (Behavioral Observation Research Interactive Software, v. 2.95, University of Torino, Torino, Italy) (34). The open-field test described by Walsh and Alstott was also used for the experimental behavioral studies (35, 36). The test indicators included the central compartment dwell time (the time from when the animal entered the experimental chamber in the open-field to when it first left the central 9-compartment), mobile times (the time the animal spent being mobile), climbing frequency (instances when the animal’s front paws left the ground or it climbed the perimeter wall), number of modifications (instances where the animal used its limbs to scratch, licked its body, and performed other grooming behaviors), the defecation and urination score (the presence of urine was scored as one, and feces were scored according to the pellet count), and the number of traverses (counting from when the animal left the central compartment for the first time and then entered again).

#### 2.3.5 Hormone level determination

On days 1, 8, and 14 of the experiment, six test animals (three females and three males) in each group were subjected to euthanasia by decapitation, blood was collected from their neck veins without disturbing the collected blood. Five milliliters of blood was collected into 10 mL centrifuge tubes, incubated at room temperature for 30 min, and then centrifuged at 4500 rpm for 15 min at 4°C. The serum was separated, transferred into 2 mL centrifuge tubes, and stored at −80°C until further use. Serum stress hormone levels, including adrenocorticotropic hormone (ACTH) and corticosterone (CORT), were quantified in plateau zokors using an enzyme-linked immunosorbent assay (ELISA) kit (Green Leaf Biotechnology Co. Ltd., Suzhou, China). Epinephrine (E) and norepinephrine (NE) were quantified in plateau zokors using an ELISA kit (Sangon Biotech Co. Ltd., Shanghai, China). The optical density (OD) of ACTH, CORT, E, and NE was measured at 450 nm (BioTek Instruments, Inc). One-way analysis of variance (ANOVA) was used to compare ACTH, CORT, E, and NE levels between the groups.

#### 2.3.6 Mortality statistics

The experimental period was divided into days 1–7 and 8–14, and 32 plateau zokor in each group were included in the mortality statistics. Animals euthanized during the study period were not included in the mortality rate. The mortality rate of the 7 days group was determined based on the animals that died naturally within the first 7 days of the experiment. The mortality rate of the 14 days group was determined by the animals that died naturally from days 8 to 14.

#### 2.3.7 Transcriptome sequencing and gene function analysis

We selected the zokors (RA = 3 and AS = 3) with the most significant decrease in stress response on the seventh day of RA and AS treatment, respectively. In addition, we randomly selected the hypothalamus of three zokors in the CON group for transcriptome sequencing. The transcriptome data pertaining to the hypothalamus of plateau zokor were obtained through sequencing performed by Guangzhou Jidio Biotechnology Co., Ltd. (Guangzhou, China). For transcriptome analysis, total RNA was isolated and genomic DNA was removed using kits following the manufacturer’s protocol (Wuhan Yunclone Technology Co., Ltd). RNA extraction, purification, cDNA library construction, transcript assembly, gene functional annotation. Differential expression analysis was performed using the DESeq2 Rpackage (v. 1.20.0) (37). To explore the underlying functions of the DEGs, we performed Gene ontology (GO) functional analyses and Kyoto Encyclopedia of Genes and Genomes (KEGG) pathway enrichment analyses of the significant DEGs. AS and the functions and pathways of genes involved in differential alternative splicing (DAS) event gene functions and pathways were analyzed for enrichment analyzed using Cytoscape (version 3.9.0) (38) (Supplementary Information S1).

#### 2.3.8 Statistical analysis

Data analysis was performed using SPSS 23.0, and one-way ANOVA was used to compare data. Specific differences between the treatment groups were determined using Duncan’s *Post-hoc* test, and differences were considered statistically significant at *p* < 0.05. Since the differences between male and female plateau zokors in the non-breeding season were not statistically significant (*p* > 0.05), the results for males and females were combined for the analysis. To establish the exclusive influence of SC on the outcomes, genes exhibiting consistent differential expression patterns before and after SC treatment in different populations of plateau zokors were excluded from the analysis. Means were compared using ANOVA, and statistical significance was set at *p* < 0.05. Duncan’s *post-hoc* test was used after the one-way ANOVA.

## 3 Results

### 3.1 Measurements of growth indicators

**Figure 1:**
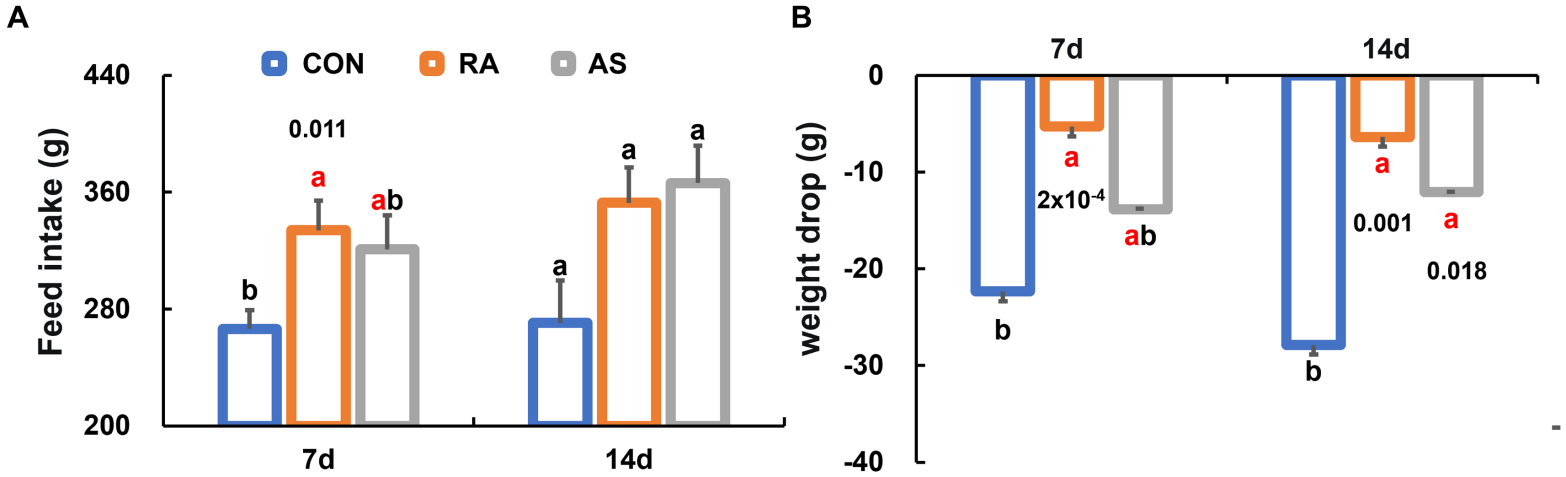
Comparison of growth performance among groups of plateau zokor. **(A)** Feed intake; **(B)** weight reduction. CON, control group; RA, *Radix astragali* group; AS, *Acanthopanax senticosus* group. Different lowercase letters indicate that under the same treatment conditions, there were significant differences between the zokor groups. The same letter indicates no significant difference between the two groups (*p* > 0.05).

We observed a significant increase in dietary intake among plateau zokors during RA treatment compared to the CON group (*p* < 0.05) (Figure 1A). Notably, the continuous efficacy of RA on dietary intake was less evident after discontinuation of the drug. Moreover, during the treatment period, animals in the RA group exhibited significantly lower weight loss compared to the CON group (*p* < 0.05). Importantly, both the RA and AS groups demonstrated a remarkable continuity of efficacy in maintaining body weight even after the drug was withdrawn (Figure 1B). Additionally, the mortality rate was highest in the CON group and lowest in the RA group after 7 days of drug intervention (Supplementary Table S1).

### 3.2 Behavioral measurements of plateau zokors

**Figure 2:**
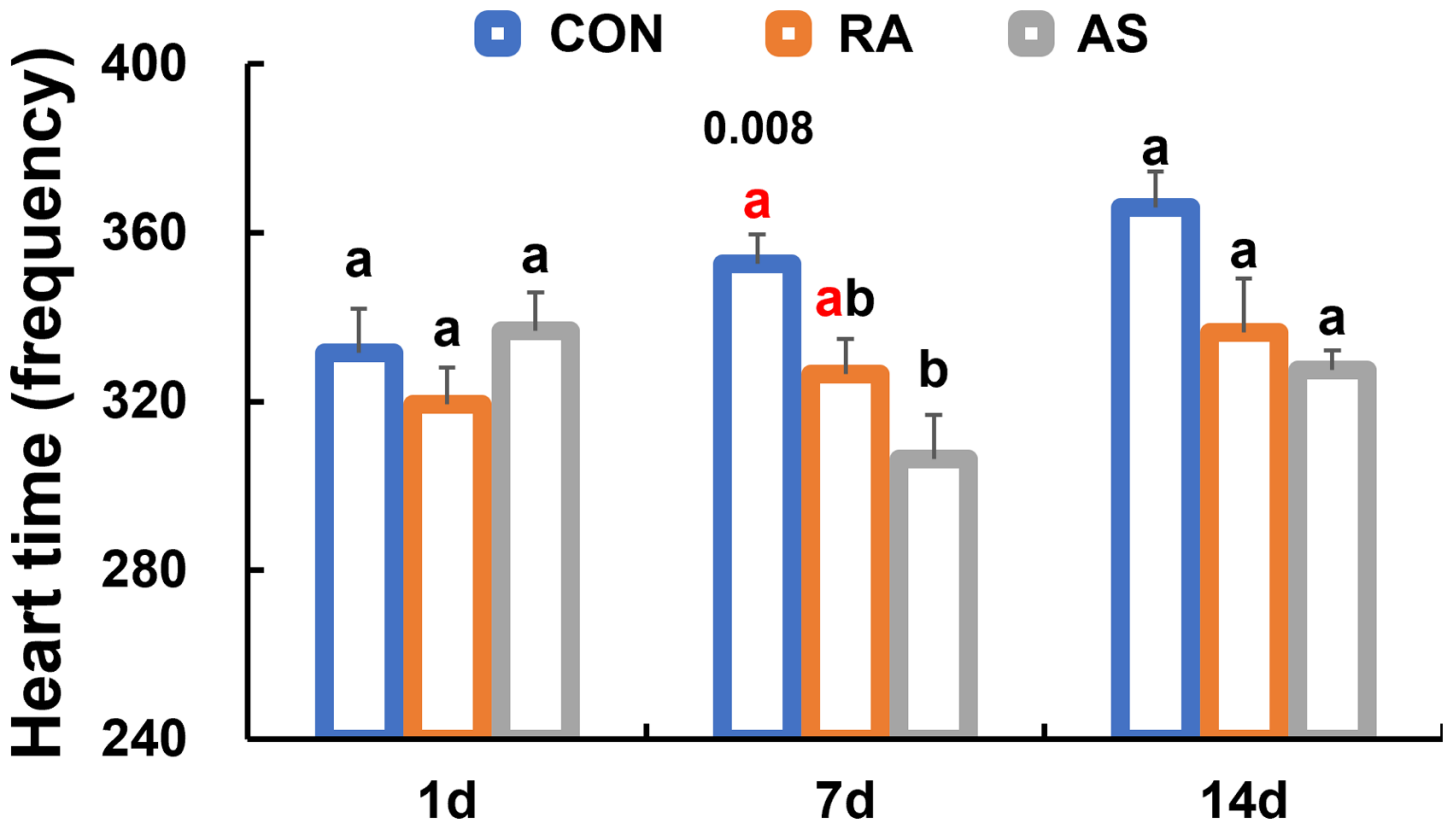
Comparison of heart rate levels of different plateau zokor groups. Different lowercase letters indicate that under the same treatment conditions, there were significant differences between the zokor groups (*p* < 0.05).

Following 1 day of treatment, there were no significant differences in heart rate among all groups (*p* > 0.05). However, after 7 days of treatment, AS significantly reduced the heart rate of plateau zokors (*p* < 0.05). Interestingly, after 14 days of treatment, there were no significant differences in heart rate among all groups (*p* > 0.05) (Figure 2).

**Figure 3:**
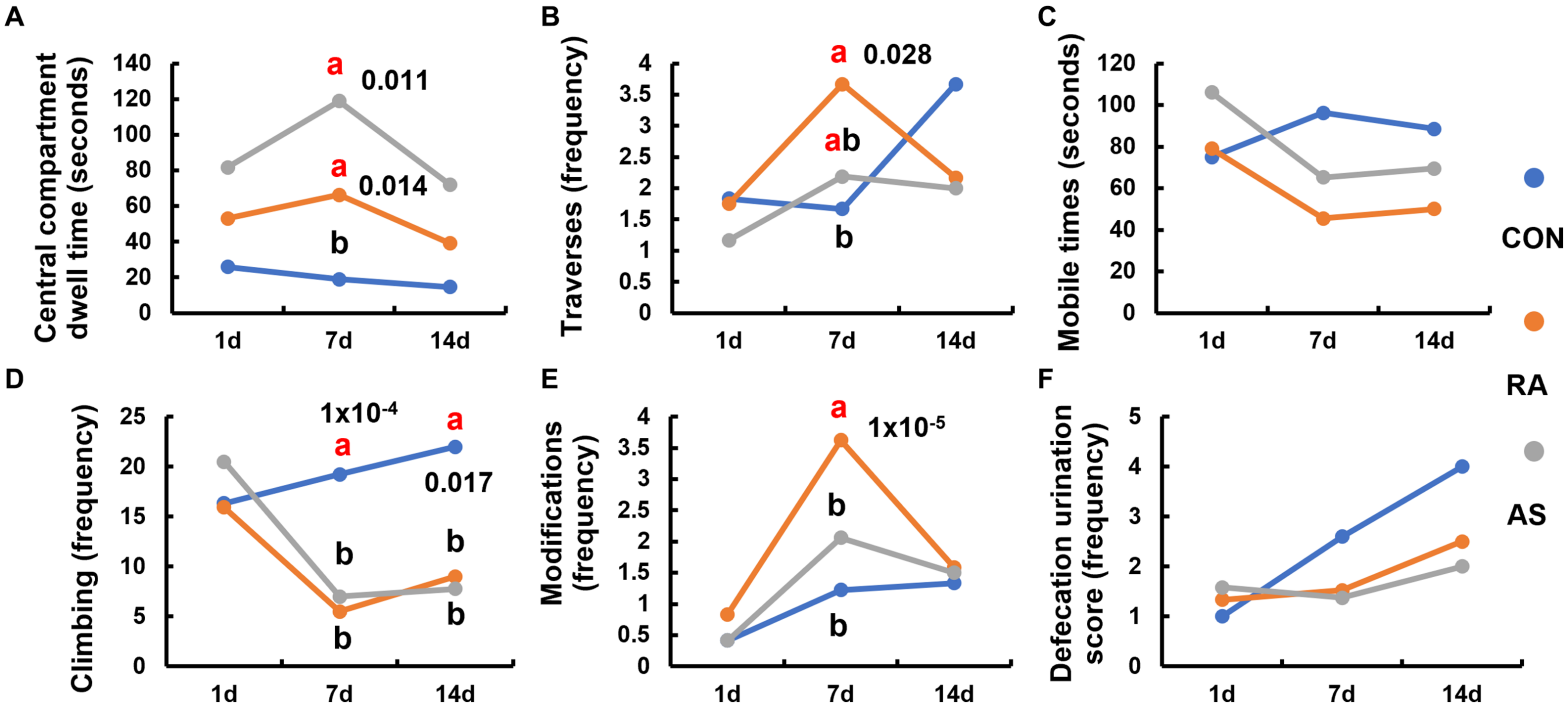
Comparison of the behavior of plateau zokor within 5 min in the open-field test. **(A)** Central compartment dwell time; **(B)** number of traverses, **(C)** mobile times; **(D)** climbing frequency; **(E)** number of modifications; **(F)** defecation and urination score. Different lowercase letters indicate that under the same treatment conditions, there were significant differences between the zokor groups (*p* < 0.05).

The stress behavior relief in plateau zokors became evident after 7 days of RA and AS treatments. The central compartment dwell time of plateau zokors in the RA and AS groups was significantly longer than that in the CON group (*p* < 0.05) (Figure 3A). Furthermore, the RA group exhibited a significantly higher number of traverses compared to the CON group (*p* < 0.05) (Figure 3B). In contrast, the mobile times of the RA and AS groups were significantly lower than those of the CON group (Figure 3C). The climbing frequency of the RA and AS groups was also significantly lower than that of the CON group (*p* < 0.05) (Figure 3D). Moreover, the number of modifications made by plateau zokors in the RA group was significantly higher than those in the CON and AS groups (*p* < 0.05) (Figure 3E). Remarkably, even after 7 days of discontinuing RA and AS treatments, the plateau zokors maintained a significantly reduced climbing frequency (*p* < 0.05) (Figure 3D). However, no significant differences were observed in central ventricular residence time, crossing frequency, activity frequency, modification frequency, and defecation or urination score. These results collectively indicate that both RA and AS effectively relieve the stress behavior of plateau zokors.

### 3.3 Comparison of stress hormones among groups

Following 7 days of treatment, the RA group exhibited significantly lower CORT hormone levels compared to the AS and CON groups (*p* < 0.05). Simultaneously, the AS group also significantly reduced CORT hormone levels in plateau zokors compared to the control group (*p* < 0.05). Furthermore, both the RA and AS groups had significantly reduced ACTH hormone levels compared to the control group (*p* < 0.05) (Table 1). NE was significantly increased in the RA and AS groups (*p* < 0.05), while there was no significant difference in E (*p* > 0.05) (Supplementary Table S2).

**Table 1:** Comparison of simultaneous CORT and ACTH hormone levels in plateau zokors.

Time	CORT (ng/mL)			ACTH (pg/mL)		
	CON	RA	AS	CON	RA	AS
**1 day**	593.27 ± 29.63^a^	602.91 ± 36.92^a^	638.51 ± 30.23^a^	230.71 ± 10.91^a^	249.46 ± 9.55^a^	258.90 ± 18.56^a^
**7 days**	542.67 ± 45.66^a^	264.46 ± 28.34^c^	408.95 ± 36.76^b^	96.05 ± 17.57^a^	63.48 ± 1.81^b^	47.99 ± 2.37^b^
**14 days**	644.01 ± 91.96^a^	418.41 ± 56.93^b^	514.97 ± 38.17^ab^	174.66 ± 27.31^a^	140.14 ± 12.91^a^	155.54 ± 28.78^a^

Statistical analyses for outcomes within groups was done using one-way. The same letter indicates no significant difference between the two groups (*p* > 0.05).

### 3.4 Transcriptome analysis of AS and RA treatment in plateau zokors

#### 3.4.1 Differentially expressed genes under AS and RA treatment

**Figure 4:**
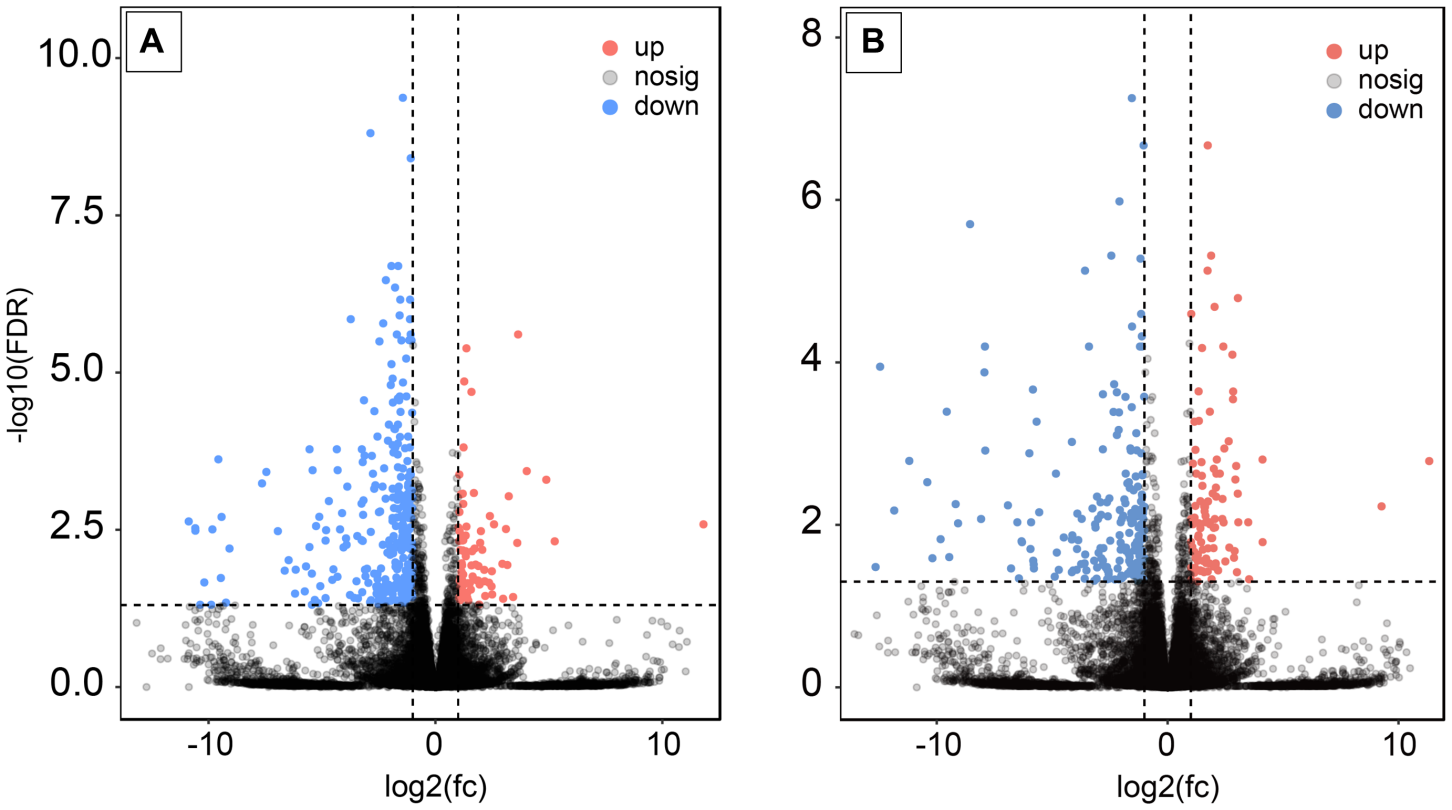
Expression analysis of DEGs after AS and RA treatments. **(A)** After AS treatment; **(B)** after RA treatment.

Following AS treatment, a total of 379 DEGs were identified, comprising 297 upregulated and 82 downregulated genes. Notably, the calponin-like transmembrane domain protein (*Clmn*) and shisa family member 6 (*Shisa6*) genes, which play a role in neuronal activity, exhibited significant upregulation by 3.1 and 3.8-fold, respectively. The DLG-associated protein 3 (*Dlgap3*) gene, associated with protein–protein interactions (PPI) and cross-chemical synaptic transmission in synapses, was significantly upregulated by 2.7-fold. Additionally, the inhibitory neurotransmitter gamma-aminobutyric acid type A receptor subunit delta (*Gabrd*) gene displayed a significant upregulation of 4.5-fold (*p* < 0.05) (Figure 4A).

Upon RA treatment, 313 DEGs were found, with 207 being upregulated and 106 downregulated. The DLG – associated protein 3 (*Dlgap3*) and potassium voltage-gated channel subfamily A regulatory beta subunit 1 (*Kcnab1*) genes, involved in PPI and cross-chemical synaptic transmission in synapses, were significantly upregulated by 2.9-fold. The family with sequence similarity 47 member E (*Fam47e*) gene, linked to myoclonic epilepsy and Parkinson’s disease, showed a notable upregulation of 2.2-fold. In contrast, the zinc finger protein 488 (*Znf488*) gene, which is associated with neuronal activity, exhibited a significant downregulation of 11.2-fold (*p* < 0.05) (Figure 4B).

#### 3.4.2 Analysis of gene ontology functions and KEGG pathways enriched by DEGs

**Figure 5:**
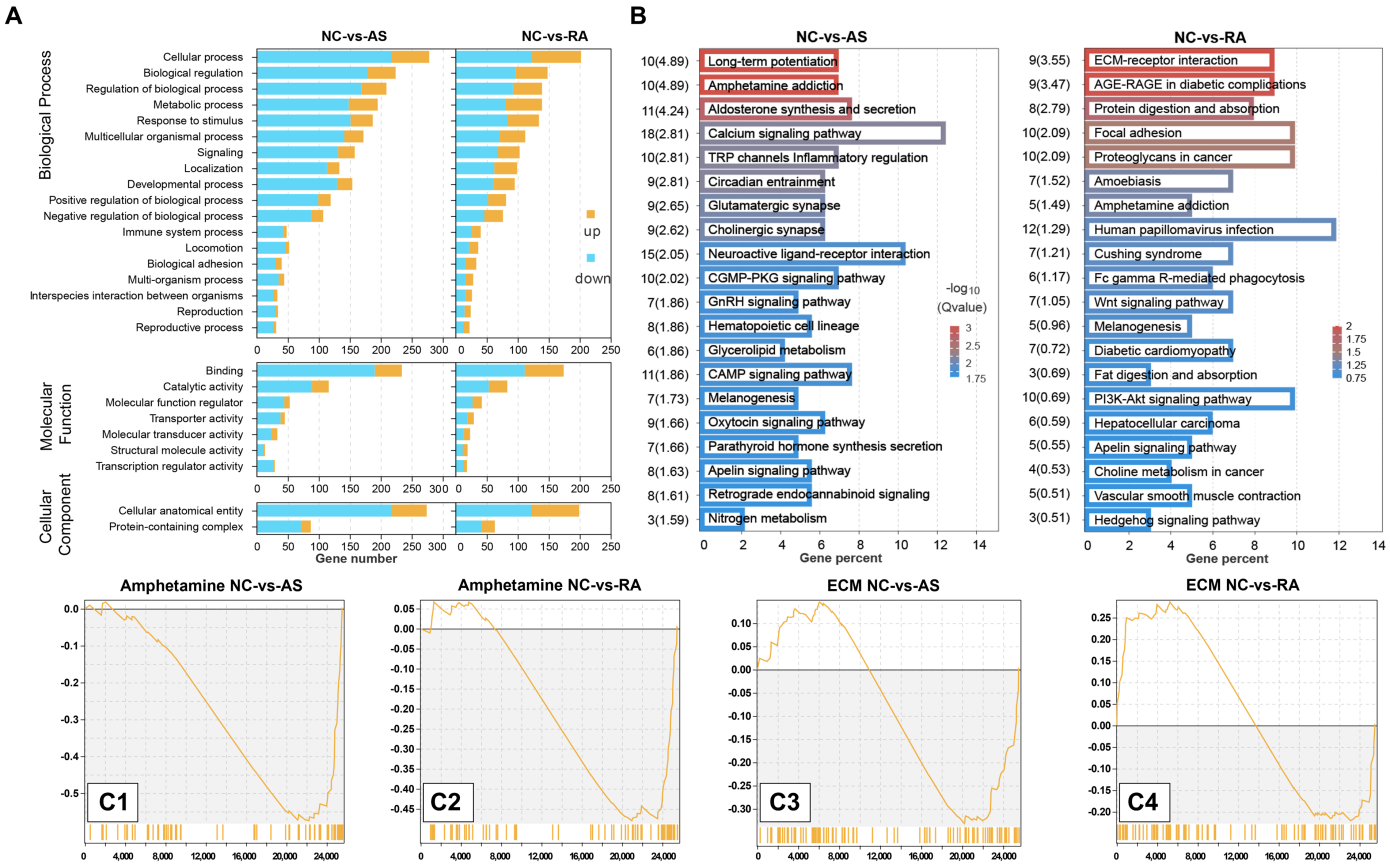
GO function and KEGG pathway of DEG enrichment. **(A)** GO function; **(B)** KEGG pathway; **(C1)** amphetamine addiction pathway after AS treatment; **(C2)** amphetamine addiction pathway after RA treatment; **(C3)** ECM-receptor interaction pathway after AS treatment; **(C4)** ECM-receptor interaction pathway after RA treatment.

We performed GO function enrichment analysis on DEGs in plateau zokors after AS and RA treatment. The GO functions enriched by DEGs following treatment with both drugs were similar, primarily encompassing cellular processes, regulation of biological processes, binding, cellular anatomical entities, and responses to stimuli (*p* < 0.05) (Figure 5A).

In the context of KEGG enrichment analysis of DEGs in plateau zokors treated with AS and RA, the significantly enriched KEGG pathways included amphetamine addiction, melanogenesis, and the apelin signaling pathway. After AS treatment, the KEGG pathways significantly enriched by DEGs encompassed retrograde endocannabinoid signaling, dopaminergic synapses, and morphine addiction. Conversely, following RA treatment, the KEGG pathways significantly enriched by DEGs included extracellular matrix (ECM)-receptor interaction, focal adhesion, and the PI3K-Akt signaling pathway (Figure 5B). It was observed that both drugs mitigated the stress response in plateau zokors by downregulating genes associated with the amphetamine addiction pathway (Figures 5C1,C2). However, the regulatory effect on the ECM receptor interaction pathway was reversed after treatment with both drugs (Figures 5C3,C4).

#### 3.4.3 Construction of PPI network and identification of key genes

To further explore the gene regulatory mechanisms linked to alleviating the stress response in plateau zokors, we conducted PPI and gene association analyses on the DEGs in plateau zokors subjected to AS treatment.

**Figure 6:**
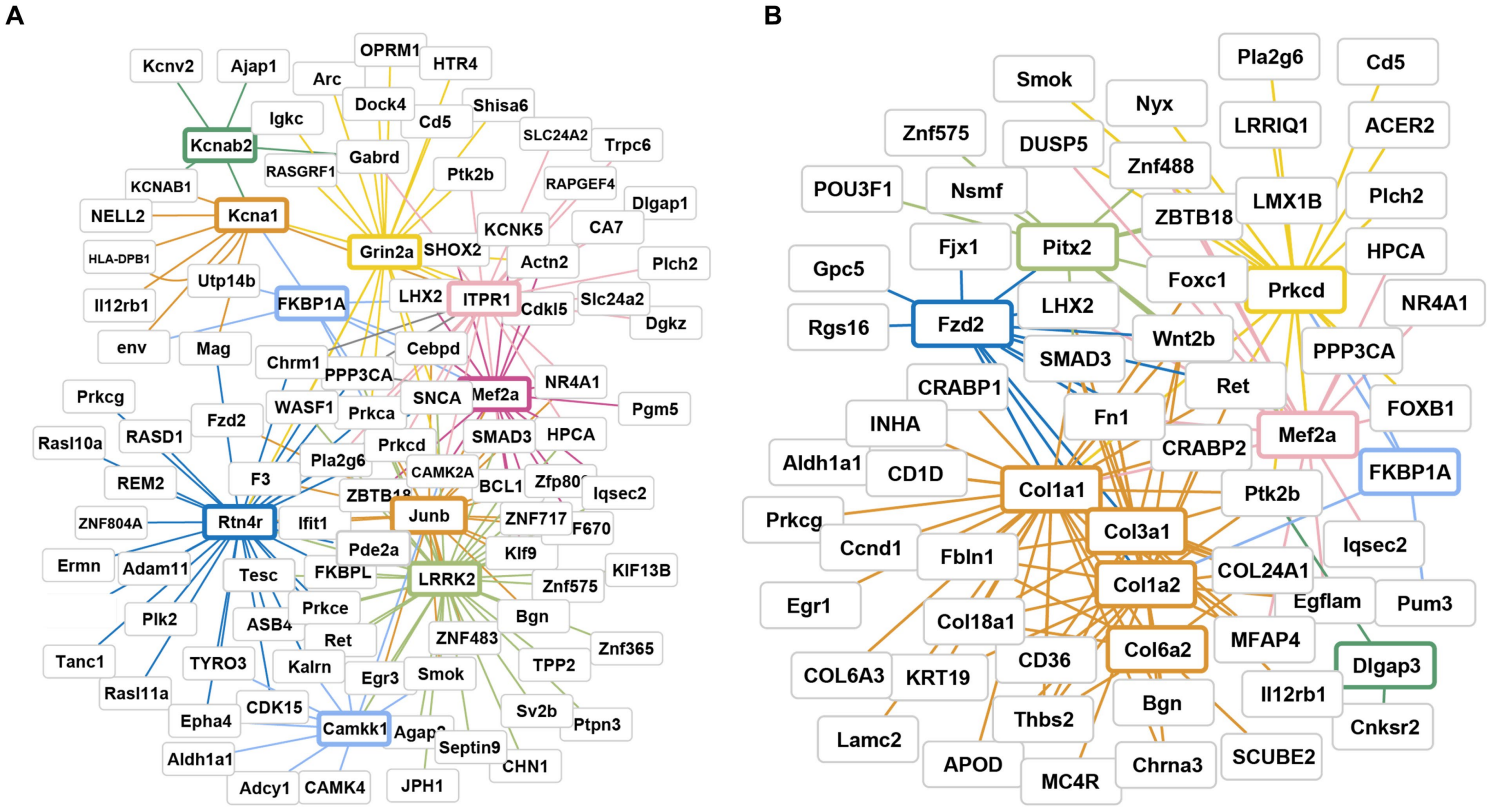
Gene interaction network and pathway analysis of DEGs after AS and RA treatment. **(A)** After AS treatment; **(B)** after RA treatment. Different colors represent different core gene family, and line color represents T10 key gene associated genes.

Following AS treatment, the PPI network included a total of 287 nodes and 1959 edges (Figure 6A; Supplementary Figure S3). We identified the top 10 genes based on the connectivity of the PPI network (Table 2). The results revealed that *Kcna1/B2*, *Grin2a*, *Itpr1*, *Mef2a*, *Lrrk2*, *Camkk1*, *Rtn4r*, and *Junb* were key genes with significantly upregulated expression, ranging from 2.0 to 3.0-fold.

**Table 2:** PPI analysis of key genes after AS and RA treatment.

Group	Gene ID	Symbol	Gene function	Fold change
AS	EOBAG001470068991/0130001991	*Kcna1/B2*	Transmembrane potassium transport to prevent neuronal overexcitation	**↑** 3.0/2.8
	EOBAG003100001991	*Grin2a*	Enhance synaptic transmission and activate ca^2+^ influx into postsynaptic cells	**↑** 2.6
	EOBAG000020227991	*Itpr1*	Mediates calcium release from endoplasmic reticulum	**↑** 2.0
	EOBAG001170003991	*Mef2a*	Neuron differentiation and stress-induced genes	**↑** 2.4
	EOBAG006070002993	*Lrrk2*	Regulating the morphology of neuronal processes in the intact central nervous system	**↑** 2.7
	EOBAG000260125992	*Camkk1*	Regulating ca^2+^ triggering signal cascade reaction	**↑** 2.2
	EOBAG000260178991	*Rtn4r*	Mediating axonal growth inhibition	**↑** 2.7
RA	EOBAG000270042494/0840039894, 03990003693/0640114991	*Col1a1/1a2, 3a1/6a2*	Constructing fibrous collagen in connective tissue to inhibit neuronal migration	**↑** 4.2/5.5, **↓** 7.0/2.65
	EOBAG000050082994	*Fkbp1a*	Immunomodulation and involvement in protein folding and transport	**↑** 2.3
	EOBAG001170003991	*Mef2a*	Neuron differentiation and stress-induced genes	**↑** 2.2
	EOBAG001130048991	*Prkcd*	Tumor suppressor factor, a positive regulator of cell cycle progression	**↓** 2.1
	EOBAG000810043991	*Fzd2*	Adjust β-chain protein dependent and independent pathways	**↓** 3.1
	EOBAG002530006991	*Pitx2*	Involved in prolactin and hormone regulating activity	**↓** 3.0

“↓” Indicates downregulation, “↑” Indicates upregulation.

After RA treatment, the PPI network consisted of 202 nodes and 1,009 edges (Figure 6B). We identified the top 10 genes based on the connectivity of the PPI network (Table 2). The results indicated that key genes such as *Col1a1*/*1a2*, *Fkbp1a*, *Mef2a*, and *Prkcd* exhibited significant upregulation, with fold changes ranging from 2.1 to 5.5. Conversely, *Col3a1*/*6a2*, *Fzd2*, and *Pitx2* genes displayed significant downregulation, with fold changes ranging from 2.65 to 7.0 (Figure 6B; Supplementary Figure S3).

## 4 Discussion

Improving animal health and mitigating the stress response in captive animals necessitate a deeper understanding of potential stress pathways. In our study, we examined the effects of RA and AS treatments on the stress response using plateau zokors as a model organism. The results revealed significant improvements in the animals’ well-being. Specifically, RA and AS treatments led to a marked increase in food intake and a reduction in weight loss, heart rate, and mortality in plateau zokors. Notably, these positive effects endured even after the cessation of drug treatment. Furthermore, these treatments were found to significantly decrease the levels of COTH and ACTH in plateau zokors, indicating a reduction in stress. Transcriptome analysis provided valuable insights into the underlying mechanisms, revealing that RA and AS treatments were associated with the downregulation of the amphetamine addiction pathway, demonstrated by their interaction with the *Camk*, *Prkcg*, and *Grin2a* gene families. Additionally, RA treatment was observed to activate the immune pathway ECM-receptor interaction, suggesting its potential in enhancing animal immune function. Overall, these findings underscore the potential of RA and AS treatments in alleviating stress and promoting animal health in captivity, while also shedding light on the complex pathways involved.

### 4.1 RA and AS treatments improved the growth performance of stressed animals

When animals are exposed to external environmental changes, it triggers the excessive ATCH production in their bodies. This, in turn, accelerates their metabolic rates and leads to the breakdown of reserves such as protein and fat, as the body attempts to generate sufficient energy to counteract the stress stimuli. Consequently, this results in a reduction in body mass (39). Our study found that both RA and AS treatments can increase food intake and reduce weight loss in plateau zokors. These findings align with Meister et al.’s research, which suggests that excessive stress negatively affects the satiety and hunger centers of the hypothalamus (40). Stress or gastrointestinal dysfunction inhibits animals’ feeding behavior, resulting in decreased food intake (41). Additionally, the study also observed a decrease in heart rate and mortality rate among plateau zokors, indicating that RA and AS administration can mitigate the elevated heart rate associated with environmental stress. This improvement in the growth performance of stressed animals enhances their adaptability to captive conditions.

### 4.2 RA and AS treatments reduced stress behavior in plateau zokors

Our behavioral experiments have shown that RA and AS treatments prolong the central compartment residence time of plateau zokors while reducing their climbing frequency. Notably, the modification frequency of RA-treated plateau zokors was significantly higher compared to the control group, aligning with the findings of Miller and Anderson (3, 42). Generally, stress tends to heighten animal agitation, causing them to seek escape from the experimental environment. Consequently, the observed decrease in climbing frequency can be attributed to reduced anxiety and stress responses. Moreover, animals in stressful situations often neglect their grooming behavior, displaying it only when they feel at ease or emotionally stable. Zhang et al. also found that Qi Bi Anshen decoction treatment significantly reduced anxiety and improved grooming behavior in depressed mice (43), which aligns with our research results. RA treatment notably reduced stress behavior in plateau zokors, as indicated by the increase in modification frequency. These findings support our hypothesis that RA and AS effectively reduce the stress response in animals and enhance their adaptability to captive environments. However, there were no significant differences in the number of movements and defecation and urination scores of plateaus zokors following RA and AS treatment. One possible explanation for this is that after drug treatment, plateau zokors tend to explore the artificially constructed experimental sites to alleviate stress reactions (44). This phenomenon is also observed in wild animals displaying exploratory and pre-adaptive behavioral characteristics toward urbanization, which may account for the lack of significant differences in movement numbers (45).

### 4.3 RA and AS treatments reduced CORT and ACTH hormone levels in plateau zokors

We discovered that RA and AS treatments significantly reduced the levels of ACTH and CORT in plateau zokors. Previous research has indicated that stress information is transmitted from the brain to the hypothalamus. This transmission activates specific neurons, leading to the production of ACTH-releasing hormone, which then travels to the pituitary gland through the pituitary portal circulation. Consequently, pituitary ACTH is synthesized and secreted, entering the bloodstream and stimulating the synthesis and release of corticosteroids (including cortisol and corticosterone) in the adrenal cortex (46, 47). ACTH plays a crucial role in regulating behavior and emotions, with moderate amounts promoting better adaptation to adverse environments. However, excessive secretion, particularly during prolonged stress, can worsen the body’s adaptive deficits (48). The binding of high levels of glucocorticoids to glucocorticoid receptors inhibits the overreaction of the HPA axis through specific pathways. This regulation helps in energy allocation, maintaining internal homeostasis, improving stress tolerance, and facilitating the body’s adaptation to adverse environments (49, 50). Consequently, RA and AS treatments have a relieving effect on the increase of stress hormones caused by environmental changes in plateau zokors. This finding aligns with Quax’s research results, which also explain the reduced stress reflexes observed in behavioral and heart rate tests conducted on plateau zokors (51). In other words, RA and AS can regulate hormone secretion in plateau zokors, maintaining internal balance under environmental stress and creating favorable physiological conditions for subsequent RA and AS treatments aimed at reducing stress responses in captive animals.

### 4.4 Effects of AS and RA on the transcriptome of the hypothalamus in plateau zokors

#### 4.4.1 AS and RA treatment alleviates stress by downregulating the amphetamine addiction pathway

Our study revealed that both AS and RA treatments significantly reduced the activity of the amphetamine addiction pathway in plateau zokors. Genes associated with this pathway were also found to be downregulated. Previous research has demonstrated that activation of the amphetamine pathway has a stimulating effect on the central nervous system, promoting wakefulness and mental alertness (52). This process relies on the release of the neuroamine amphetamine (APA), which is regulated by the presynaptic membrane CaMK family (CaMK2, CaMKK1) and PRKCG enzymes. Additionally, the Grin2a enzyme regulates the activation of the postsynaptic membrane by APA (53, 54). The downregulation of these three enzymes disrupts the excitatory process of neurons and inhibits the release of central norepinephrine, resulting in a weakened inhibitory response of ACTH and cortisol to acute stress (55, 56). This could explain the significant decrease in ACTH levels observed after AS and RA treatment. Similar findings have been reported in studies investigating negative feedback inhibition using synthetic glucocorticoid drugs. These studies have shown that reducing the level of FKBP protein contributes to the negative feedback of glucocorticoids on the HPA axis, leading to the inhibition of the *Pomc* gene expression in pituitary adrenocorticotropic hormone cells and affecting neuronal excitatory processes (57, 58). Our observations suggest that the stress relief observed in plateau zokors following AS and RA treatment may be attributed to the inhibition of the amphetamine addiction pathway. Therefore, we propose that the mechanism by which AS and RA treatments regulate animal stress may primarily involve interfering with the transport and binding abilities of the presynaptic and postsynaptic membranes, ultimately reducing the stress response in animals.

#### 4.4.2 RA exhibits additional immune-enhancing effects in relieving stress response

Our study observed that RA treatment, compared to AS treatment, enriched the ECM-receptor interaction pathway and significantly upregulated the expression of *Col1a1*/*1a2*. Previous reports have demonstrated that *Col1a1/1a2* upregulation plays a crucial role in alleviating cardiac burden caused by adrenaline elevation, as demonstrated in a study on alleviating adrenaline-induced heart failure in mice (59). This finding may explain the significant downregulation of CORT observed in plateau zokors after 7 days of RA treatment. Notably, PPI enrichment analysis revealed that the *Col1a1/1a2* gene, enriched in the ECM-receptor interaction pathway, is closely associated with immune cell-related genes such as *CD36* and *Ccnd1*. This suggests that RA treatment may improve the immune function of plateau zokors. A study by Xie et al. also demonstrated that RA treatment significantly improves immune function in patients with neuroinflammatory diseases (60), aligning with our findings. This phenomenon may be attributed to the regulatory effects of *Col* family genes on cell activity and inflammation, leading to immune system activation (61). The improvement in immune function, in turn, negatively regulates the inflammatory response and plays a crucial role in alleviating animal stress response during environmental changes, thereby maintaining overall body health. Further research on this immune enhancement will contribute to a better understanding of how RA treatment alleviates animal stress response.

## 5 Conclusion

The domestication of animals plays a crucial role in enhancing the production and quality of animal products, thereby contributing to sustainable agricultural ecological development, and ensuring food safety. Moreover, the captive propagation of wild animals is an essential component of breeding plans and conservation efforts aimed at safeguarding endangered species. Recent research has shed light on the potential of medicinal plants, such as those used in Traditional Chinese Medicine, in mitigating animal stress. In this regard, RA and AS have emerged as intriguing avenues for investigating the mechanisms underlying their anti-stress effects. This study focuses on evaluating the impact of RA and AS treatment on plateau zokors in captive environments by examining changes in body weight, heart rate, behavior, stress hormone levels, and the hypothalamic transcriptome. The findings convincingly demonstrate that these treatments effectively alleviate stress responses in plateau zokors and regulate their stress processes through the amphetamine addiction pathway. Consequently, the utilization RA and AS to alleviate stress in captive animals emerges as a straightforward and efficacious approach. However, our comprehension of the regulation of this stress response remains incomplete, necessitating further research on neuronal differentiation and the metabolic pathways of RA and AS. Such investigations will provide valuable insights for developing strategies to alleviate animal stress under captive conditions.

## Data availability statement

The datasets presented in this study can be found in online repositories. The names of the repository/repositories and accession number(s) can be found at: https://www.ncbi.nlm.nih.gov/bioproject/?term=PRJNA1014125.

## Ethics statement

The animal studies were approved by The Animal Ethics Committee of Gansu Agricultural University approved the experimental procedure, and the local authorities approved the research plan (GAU-LC-2020-014), and conducted in compliance with the ARRIVE guidelines. The studies were conducted in accordance with the local legislation and institutional requirements. Written informed consent was obtained from the owners for the participation of their animals in this study.

## Author contributions

FZ: Data curation, Formal analysis, Investigation, Methodology, Resources, Writing – original draft. YT: Formal analysis, Resources, Writing – original draft. ZC: Resources, Writing – review & editing. KA: Investigation, Methodology, Writing – original draft. YL: Methodology, Resources, Writing – original draft, Writing – review & editing. JS: Conceptualization, Formal analysis, Funding acquisition, Investigation, Project administration, Resources, Supervision, Writing – original draft, Writing – review & editing.
