# Supplementary material for: Two plants improve stress response of a subterranean herbivore by downregulating amphetamine addiction pathways
Source: Front Vet Sci. 2024 Jan 12;10:1342630. doi: 10.3389/fvets.2023.1342630 (PMC10811048; doi:10.3389/fvets.2023.1342630)
Supplement: Supplementary file 1 [file Data_Sheet_1.docx]

Supplementary Material

**Two plants improve stress response of a subterranean herbivore by downregulating amphetamine addiction pathways**

Feiyu Zhang^1^, Yuchen Tan^2,3^, Zhiyuan Cai^1^, Kang An^2,3^, Yongjie Liu^1*^ and Junhu Su^2,3*^

1 Southwest Survey and Planning Institute of National Forestry and Grassland Administration, Kunming 650031, China

2 College of Grassland Science, Key Laboratory of Grassland Ecosystem (Ministry of Education), Gansu Agricultural University, Lanzhou 730070, China

3 Gansu Agricultural University-Massey University Research Centre for Grassland Biodiversity, Gansu Agricultural University, Lanzhou 730070, China

***** Correspondence: Dr. Yongjie Liu, liuyongjie203@163.com Tel. +86-871-65637161, Fax: + 86-871-65637161; Dr. Junhu Su, sujh@gsau.edu.cn. Tel. +86-931-7631213, Fax: +86-931-7631227.

**Supplementary Materials**

**Total RNA extraction**

Total RNA was extracted from the hypothalamus of plateau zokor using TRIzol reagent (Invitrogen Carlsbad, CA, USA) according to the manufacturer’s protocol. RNA integrity was assessed using the RNA Nano 6000 Assay Kit of the Bioanalyzer 2100 system (Agilent Technologies, CA, USA). Sequencing was performed at Guangzhou Jidio Biotechnology Co., Ltd (Guangzhou, China) (Yang et al., 2015).

**Library preparation for transcriptome sequencing**

Briefly, mRNA was purified from total RNA using poly-T oligo-attached magnetic beads. Fragmentation, cDNA, and library preparation synthesis were then performed (Yang et al., 2015).

**Clustering and sequencing**

Clustering of index-coded samples according to the manufacturer’s instructions (Yang et al., 2015).

**Sequence alignment and functional analysis**

Raw reads in the FASTQ format were processed and cleaned (clean reads) using Trinity (version 2.4.0). The RNA-seq reads in the FASTQ file were mapped to the Upper Galilee mountains blind mole rat (*Nannospalax galili*) reference genome (BioProjects: PRJNA254049). After the raw read data were cleaned, Trinity was used for transcript assembly, Corset (Nadia M Davidson Alicia Oshlack 2014) was used to aggregate redundant transcripts, and the assembled transcripts were evaluated using tblastn, augustus, and hmmer software. Gene function was annotated using the Nr, Nt, Pfam, KOG/COG, Swiss-Prot, KEGG, and GO databases (Kanehisa et al., 2019; Kanehisa et al., 2023). Differential expression analysis was performed using the DESeq2 R package (version 1.20.0) (Yang et al., 2015). We used the native version of GSEA analysis tool http://www.broadinstitute.org/gsea/index.jsp. GSEA was performed using GO, KEGG datasets, respectively. PPI analysis of differentially expressed genes was based on the STRING database, which is known and predicts protein-protein interactions.

**Real-time Quantitative polymerase chain reaction qPCR**

Liver tissues from eight plateau zokors were selected for qPCR analysis within the CON, RA, and AS group, respectively. Following the operating instructions of the RNAiso Plus Total RNA Extraction Kit, total RNA was extracted. Reverse transcription was performed utilizing the Evo M-MLV RT Premix for qPCR kit (Accurate, Changsha, China). The cDNA was subjected to qPCR amplification utilizing TB Green® Premix Ex Taq™ II (Takara, Beijing, China) in a real-time PCR system (Light Cycler 96 System, Roche). To interpret the qPCR outcomes, the 2−ΔΔCq method was applied to deal with the results of qPCR, and the relative expression level of each gene was corrected using the reference gene Primer sequences were synthesized employing a commercial sequencing system (TsingKe, Xi’an, China), the details of which can be found in Table S3.

Yang M, Zhu LP, Pan C, Xu LM, Liu YL, Ke WD, Yang PF. Transcriptomic analysis of the regulation of rhizome formation in temperate and tropical lotus (*Nelumbo nucifera*). *Sci Rep*. (2015) 17:13059.

Kanehisa M. Toward understanding the origin and evolution of cellular organisms. *Protein Sci*. (2019) 28:1947-1951.

Kanehisa M, Furumichi M, Sato Y, Kawashima M, Ishiguro-Watanabe M. KEGG for taxonomy-based analysis of pathways and genomes. *Nucleic Acids Res*. (2023) 51:D587-D592.


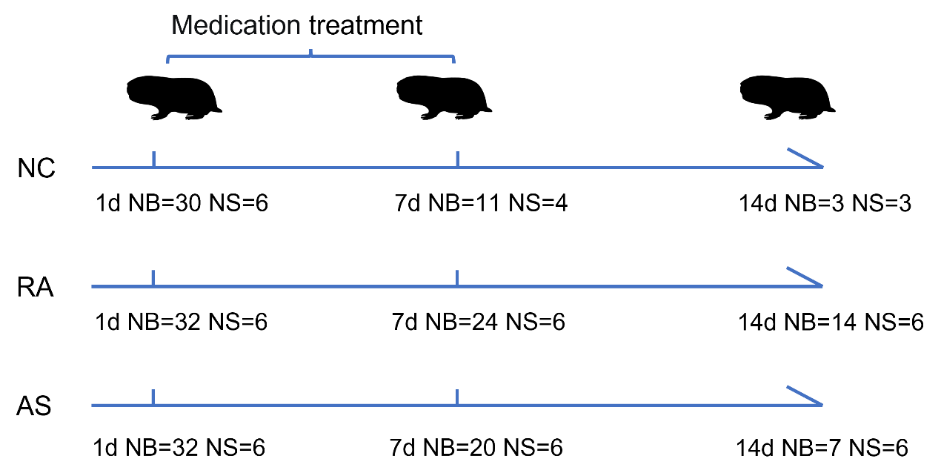


**Figure S1.** Experimental treatment mode. The first day of the behavioral experiment was followed by medication. NB, number of animals in behavioral experiments. NS, the hormone measures the number of experimental animals.


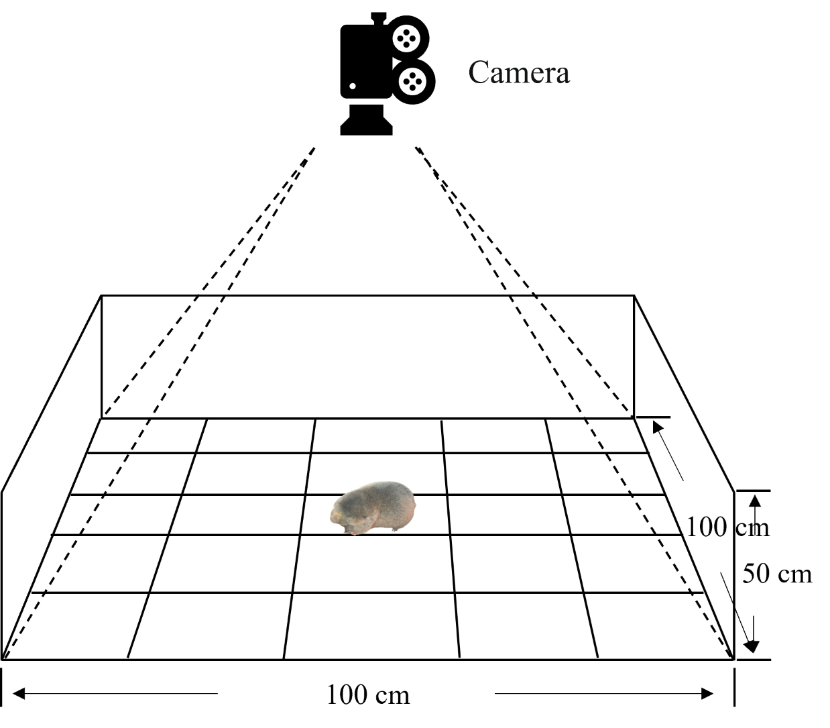


**Figure S2.** Open field test box. Open field test box for width 100×100×50 cm high glass box, box around with black sticker shade, experiment of division at the bottom of 25×20 cm square, for the case in the middle of the nine central frames, the remaining 16 case as the peripheral area. Using infrared video webcam to record behavior.


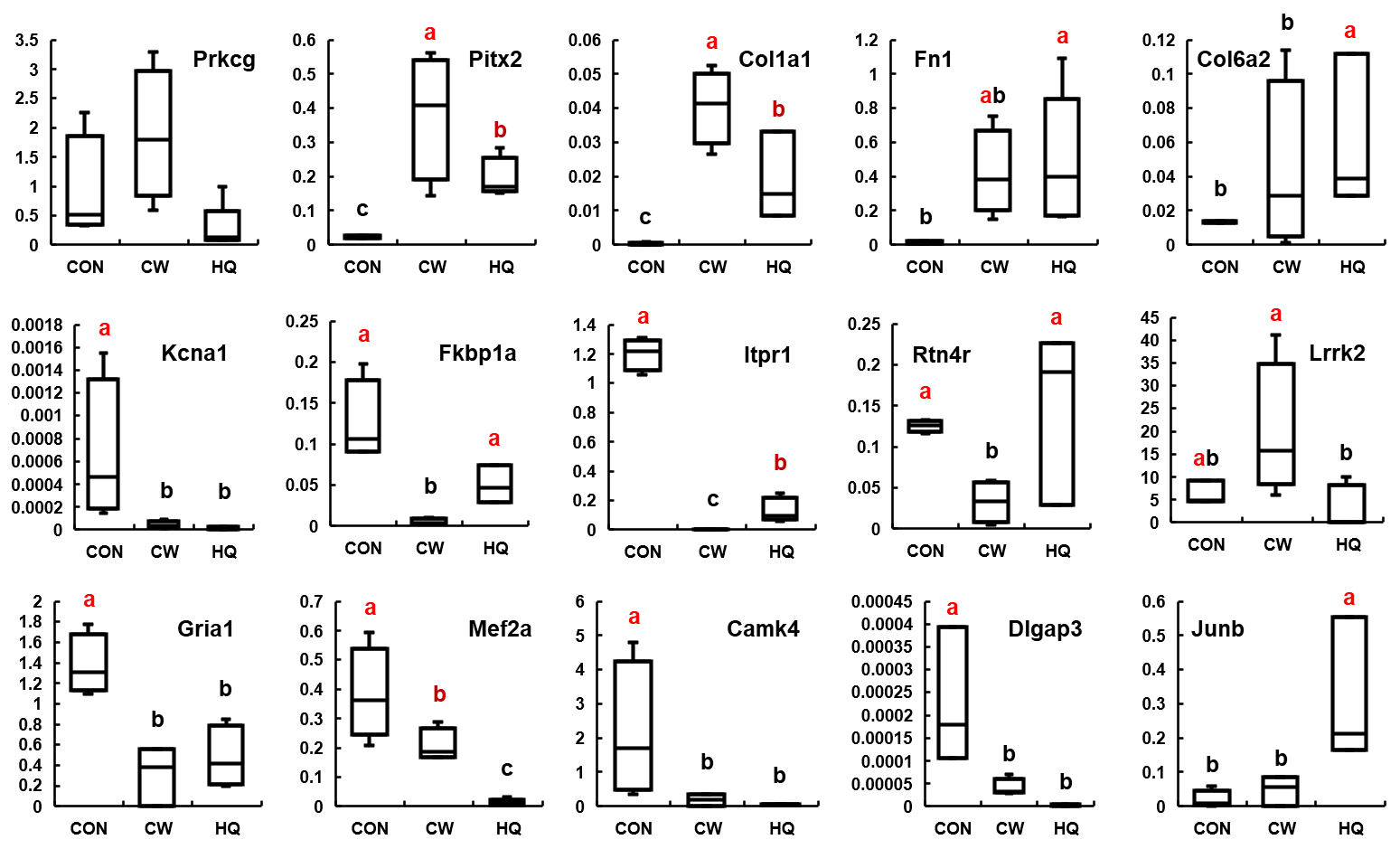


**Figure S3.** Validation by RT-qPCR. The same letter indicates no significant difference between the two groups (*P* > 0.05).

Table S1. Mortality of plateau zokors after 7 and 14 days.

| Time | Mortality rate % | | |
| --- | --- | --- | --- |
|  | CON group (*n* = 32) | RA (*n* = 32) | AS (*n* = 32) |
| 7 d | 46.86 | 15.62 | 34.38 |
| 14 d | 68.75 | 31.25 | 40.63 |

**Note:** CON, Control group; RA, *Radix astragali* group; AS, *Acanthopanax senticosus* group.

**Table S2.** Comparison of simultaneous E and NE hormone levels in plateau zokors

| **Time** | **E (ng/mL)** | | |  | | **NE (ng/mL)** | | |
| --- | --- | --- | --- | --- | --- | --- | --- | --- |
|  | **CON** | **RA** | **AS** |  | | **CON** | **RA** | **AS** |
| **1 d** | 1.21 ± 0.28^a^ | 1.13 ± 0.25^a^ | 0.82 ± 0.11^a^ | | 1.43 ± 0.38^b^ | | 1.23 ± 0.19^b^ | 1.16 ± 0.52^a^ |
| **7 d** | 1.11 ± 0.15^a^ | 0.77 ± 0.06^a^ | 1.19 ± 0.24^a^ | | 1.79 ± 0.21^b^ | | 9.51 ± 3.48^a^ | 3.61 ± 1.11^a^ |
| **14 d** | 1.19 ± 0.20^a^ | 0.73 ± 0.10^ab^ | 0.62 ± 0.15^b^ | | 8.57 ± 3.61^a^ | | 11.02 ± 4.43^a^ | 4.28 ± 0.39^a^ |

**Note**: Epinephrine (E); norepinephrine (NE). Statistical analyses for outcomes within groups was done using one-way. The same letter indicates no significant difference between the two groups (*P* > 0.05).

**Table S3.** List of the qPCR primer sequence.

| **Gene Symbol** | **Primer Sequence (5′→3′)** | **Product Size (bp)** |
| --- | --- | --- |
| Kcna1 | AGGAACAGAAGAGCTATATGCCA  CTTGTCGAGCAGGGCAAGTA | 134 |
| Fkbp1a | TGATCGGTCTTAATCCCGGC  CGCCTCCCGGTTCTTCTTTT | 186 |
| Camk4 | CTGGTCCCCAAGGTAAGCTG  CTTGTGGGAGGCAGATGTGT | 149 |
| Gria1 | CACCACAACCACATGGAGGA  CAATGCAGCGTTAGTCGTCG | 150 |
| Itpr1 | GCCAAGAACCGTCTCCAAGT  TGGGCCTCCTCAGCATTTAC | 132 |
| Mef2a | ACAACATCCCGGTGTCATCC  GGACAGTGAGCTGGATTGCT | 134 |
| Junb | CACTTGCCCTCACCTGTCTT  CCTGGGTGTTGGAGCAGAAT | 137 |
| Rtn4r | TTCCCAATCAGTGCTGGCAT  GTAGTGTGGCGGAGAGAAGG | 200 |
| Lrrk2 | TGCCCCATTGATGGATGCTT  TGCGATCACAGCACACTCAT | 197 |
| Col1a1 | CGAATACTTGCGCCAACAGG  ACATCACGTCATCGCAGAGG | 159 |
| Pitx2 | GATGGCCAAGAAAAGGACGC  GTGCTCCTCTCCCAGGAAAC | 159 |
| Col6a2 | CCATCCGACTGGACGATGAG  GAGCTGGTTGTAGGCGAACT | 180 |
| Dlgap3 | TACTTCGGTGACCCATTGGC  ACAGCTCGTTAGTGCTGCTT | 123 |
| Prkcg | CCTGAGAAGGTGTTCACGGT  AGACAACAGCCAGAATCAAGGT | 110 |
| Fn1 | TCAGGACCCTGAGGATAGCA  AGGGAATTGAGCACAACTTGGA | 199 |
